# Relationships between muscle sympathetic nerve activity and novel indices of arterial stiffness using single oscillometric cuff in patients with hypertension

**DOI:** 10.14814/phy2.15270

**Published:** 2022-05-19

**Authors:** Hiroyuki Sugimoto, Takuto Hamaoka, Hisayoshi Murai, Tadayuki Hirai, Yusuke Mukai, Takashi Kusayama, Shinichiro Takashima, Takeshi Kato, Shigeo Takata, Soichiro Usui, Kenji Sakata, Masa‐Aki Kawashiri, Masayuki Takamura

**Affiliations:** ^1^ 38301 Department of Cardiovascular Medicine Kanazawa University Graduate School of Medical Sciences Kanazawa Japan; ^2^ Penn State Heart and Vascular Institute Pennsylvania State University College of Medicine Hershey Pennsylvania USA; ^3^ Kanazawa Municipal Hospital Kanazawa Japan

**Keywords:** arterial pressure‐volume index, arterial stiffness, arterial velocity pulse index, hypertension, muscle sympathetic nerve activity

## Abstract

The arterial velocity pulse index (AVI) and arterial pressure‐volume index (API) have been proposed as new arterial stiffness indices that can be measured using an oscillometric cuff. Sympathetic nerve activity (SNA) contributes to arterial stiffness via increasing vascular smooth muscle tone. However, the associations between SNA and the AVI or API are not understood. The purpose of this study was to evaluate the relationships between muscle sympathetic nerve activity (MSNA) and the AVI or API in healthy individuals and patients with hypertension (HT). Forty healthy individuals (40.1 ± 15.2 years, 8 females) (healthy group) and 40 patients with HT (60.2 ± 13.6, 18 females) (HT group) were included in this study. The AVI, API, MSNA, beat‐by‐beat blood pressure, and heart rate were recorded simultaneously. The AVI and API were higher in the HT group than in the healthy group (AVI, 26.1 ± 7.6 vs. 16.5 ± 4.0, *p* < 0.001; API, 31.2 ± 8.6 vs. 25.5 ± 7.2, *p* = 0.002). MSNA in the HT group was also higher than in the healthy group (*p* < 0.001). MSNA was correlated with the AVI, but not with the API, in both the healthy group (*R* = 0.52, *p* = 0.001) and HT group (*R* = 0.57, *p* < 0.001). MSNA was independently correlated with the AVI in multivariate analysis (*ß* = 0.34, *p* = 0.001). In conclusion, AVI, obtained by a simple and less user‐dependent method, was related to the MSNA in healthy individuals and patients with HT.

## Graphical abstract

Arterial velocity pulse index (AVI) is a novel arterial stiffness index that can be measured by a single oscillometric cuff. We found that AVI was significantly correlated with muscle sympathetic nerve activity (MSNA) in both healthy subjects and patients with hypertension, which is likely related to the influence of MSNA on arterial wave reflection.

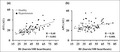

**Abbreviations**
AI: augmentation index
API: arterial pressure‐volume index
AVI: arterial velocity pulse index
Ba: brachial‐ankle
BF: burst frequency
BI: burst incidence
BP: blood pressure
Cf: carotid‐femoral
CV: cardiovascular
DBP: diastolic blood pressure
HT: hypertension
MSNA: muscle sympathetic nerve activity
PWV: pulse wave velocity
SBP: systolic blood pressure

## 1 INTRODUCTION

Arterial stiffness is a well‐known predictor of cardiovascular (CV) events and mortality (Mitchell et al., 2010; Vlachopoulos et al., 2010). Several methods have been developed to measure arterial stiffness (Laurent et al., 2006). Pulse wave velocity (PWV) is an established non‐invasive method of assessing arterial stiffness by calculating flow velocity between two points (e.g., between carotid and femoral/brachial and ankle) (Laurent et al., 2006). The carotid and femoral (cf)‐PWV value is an independent predictor of CV events in patients with HT (Boutouyrie et al., 2002; Laurent et al., 2001, 2003) and has been regarded as the gold standard method to measure arterial stiffness (Laurent et al., 2006). However, inaccurate measurement of the distance between recording sites compromises the PWV data (Chiu et al., 1991; Huybrechts et al., 2011), and the presence of morphological variations in vessels/body shape (e.g., atherosclerosis and obesity) affects accuracy (Van Bortel et al., 2002). Augmentation index (AI) has been reported as another index of arterial stiffness (Laurent et al., 2006; O'Rourke & Mancia, 1999). The AI is calculated from arterial waveforms and is widely recognized as an index of arterial reflected waves (Laurent et al., 2006). The AI is obtained non‐invasively by applying a manometry device to a central (carotid) or peripheral (radial) artery (Laurent et al., 2006), but well‐trained investigators are required to measure waveforms adequately, (Elliot et al., 2020) and some technical limitations of the measurement (e.g., the effects of obesity and aging) have been suggested (Laurent et al., 2006). Therefore, the use of cf‐PWV and AI remains difficult in daily clinical practice for many institutes.

The arterial velocity pulse index (AVI) and arterial pressure‐volume index (API) are new arterial stiffness indices (Komine et al., 2012; Sueta et al., 2015), which can be obtained simultaneously by applying an oscillometric blood pressure (BP) cuff to the left upper arm (Komatsu et al., 2017; Komine et al., 2012; Sueta et al., 2015). The pulse wave measured from the cuff was automatically differentiated into forward and reflected waves (Nas‐1000 system; Nihon Koden, Nishiochiai Shinjuku‐Ku, Japan). Arterial velocity pulse index is devised as an index of arterial reflected waves (Komatsu et al., 2017; Sueta et al., 2015), and AVI correlates with AI, another index of reflected waves (Komatsu et al., 2017). The API is an index calculated from the pressure‐volume curve of the upper limb artery and was related to conventional indices of arterial stiffness, as well as carotid‐femoral (cf‐) and brachial‐ankle (ba‐) pulse wave velocities (PWV) (Komatsu et al., 2017; Komine et al., 2012). Because of the simple (requiring only 2 min to obtain values) and less user‐dependent method, AVI and API are promising techniques to quantify indices of arterial stiffness, which can be applied widely in daily clinical practice. Indeed, the clinical usefulness of these parameters in patients with coronary artery diseases (Sueta et al., 2015), and patients with end‐stage renal diseases were reported (Sueta et al., 2015). However, so far, the detailed characteristics of these new indices have not been fully verified.

In addition to structural remodeling of the artery, sympathetic nerve activity (SNA) is recognized as an important contributor to arterial stiffness via changing the artery smooth muscle tone (Bank et al., 1995; Nardone et al., 2020). Elevated SNA is also an independent predictor of CV disease like heart failure (Julius, 1993) and is associated with disease severity (Barretto et al., 2009; Grassi et al., 2018). Many physiological studies have applied muscle sympathetic nerve activity (MSNA) as a parameter of postganglionic SNA innervating resistance vessels (Floras, 2009; Wallin & Charkoudian, 2007). Indeed, MSNA can affect arteriole smooth muscle tone. Thus, significant associations between MSNA and arterial stiffness indices have been reported (Casey et al., 2011; Hart et al., 2013; Holwerda et al., 2019; Millar et al., 2019; Swierblewska et al., 2010). However, the relationship between SNA and the AVI or API remains unclear. Additionally, the AVI and API differences between healthy individuals and patients with hypertension (HT) are not thoroughly understood. Evaluating arterial stiffness is crucial in treating HT and predicting the outcome (Laurent et al., 2006), then to use AVI and API efficiently in clinical practice, to understand the detailed character of these indices in patients with HT is important. Therefore, the purpose of this study was to evaluate the relationships of MSNA with the AVI and API in healthy individuals and patients with HT. We also examined the differences in the AVI and API between healthy individuals and patients with HT. We hypothesized that AVI and API are correlated with MSNA, and these values are higher in patients with HT than in healthy individuals.

## 2 METHODS

### 2.1 Subjects

Forty healthy participants (healthy group) and 40 patients with HT (HT group) were consecutively enrolled in this study. HT was defined as systolic BP (SBP) ≥140 mmHg, diastolic BP (DBP) ≥90 mmHg (confirmed at least twice different visits of >1week interval) (Umemura et al., 2019), and/or previous diagnosis and treatment of HT. HT patients attending Kanazawa university hospital or Kanazawa municipal hospital were recruited as already diagnosed HT patients. If healthy subjects met the above HT criteria, they were classified into the HT group. All healthy participants had received an annual medical check‐up, and no diseases had been detected. None of the healthy participants took any medication or supplements. Only patients with essential HT were included in the HT group; patients diagnosed with secondary HT were excluded. HT patients with other active CV diseases (i.e., symptomatic cerebral/coronary artery diseases or heart failure) and/or severe renal dysfunction (estimated glomerular filtration rate <30 mL/min/1.73 m^2^) were also excluded. Fifteen patients with HT had previously received antihypertensive medications, while the participants in the healthy control group did not take any medications. This study protocol was approved by the Research Ethics Board of Kanazawa University (Kanazawa, Japan) and conformed with the Declaration of Helsinki. This study was registered in the University Hospital Medical Information Network Center (Tokyo, Japan) Clinical Trials Registration System (UMIN000036722). All participants provided written informed consent. The datasets generated during and/or analyzed during the current study are not publicly available but are available from the corresponding author on reasonable request.

### 2.2 Measurements

Participants were asked to abstain from alcohol, caffeine, and strenuous exercise for 24 h before the experiment. The subjects were requested to take a light meal in the morning to avoid hunger and satiety. The subjects entered the laboratory after voiding. Beat‐by‐beat BP, heart rate (HR), and MSNA were recorded simultaneously in a resting supine position. After an acclimation period (at least 5 min and until the BP, HR, and MSNA became stable), the data were collected in a supine position for at least 5 min. Beat‐by‐beat BP was continuously recorded from the radial artery using a non‐invasive tonometry monitoring system (JENTOW‐7700; Nihon Colin, Hiroshima, Japan). The AVI and API were measured simultaneously using the Nas‐1000 system (Nihon Koden, Nishiochiai Shinjuku‐Ku, Japan) after the acclimation period. The average value of the two measurements was used as the AVI and API of each individual. The AVI and API were calculated as described previously (Komine et al., 2012; Sueta et al., 2015). A dedicated oscillatory cuff was applied to the left upper arm and inflated to supra‐SBP. The time series of the cuff pressure and the amplitude of the pulse oscillations were recorded by the device (Nas‐1000). The device automatically detected two peaks of the waveform, and the first peak was defined as an incident wave (P1), and the second peak was defined as a reflected wave (P2) (Sueta et al., 2015). The obtained oscillometric waveforms were time differentiated, then the first peak of the differentiated waveform (Vf) followed by the nadir of the differentiated waveform (Vr) were acquired. The AVI was calculated as 20 × Vr/Vf (arbitrary unit, AU) (Sueta et al., 2015). Therefore, a higher AVI indicates a higher reflected wave velocity likely caused by stiffened artery walls (Komatsu et al., 2017; Sueta et al., 2015). Using the same data of the time series of the cuff pressure and the amplitude of the pulse oscillations used for the AVI, the pressure‐volume curve was constructed by the numerical integration of the local slopes. The entire pressure‐volume curve was fitted using an equation, and a numerical coefficient of the equation was defined as API (Komine et al., 2012). All calculations for AVI/API were performed automatically by the device (Nas‐1000 system) for approximately 2 min in each measurement, and no manual adjustments were needed. MSNA was recorded from the left peroneal nerve using a tungsten microelectrode as described previously (Hamaoka, Murai, et al., 2021; Inomata et al., 2014; Murai et al., 2009). After pulse‐synchronous sympathetic bursts were obtained percutaneously, the electrodes were connected to a preamplifier at a gain of 1000 and to an amplifier at a gain of 70. The signals were band‐pass filtered (500–3000 kHz), and a resistance‐capacitance integrated circuit was used with a time constant of 0.1 s using the PowerLab recording system (Model ML 785/85P; ADI Instruments, Bella Vista, NSW, Australia). The raw nerve signal was obtained at 12 kHz. Other signals were obtained at 1000 Hz. MSNA bursts were detected as described in prior reports (Cui et al., 1985, 2006; Hamaoka, Blaha, et al., 2021). Briefly, first, MSNA bursts in the integrated MSNA traces were identified by visual inspection of the data, together with the burst sound from the audio amplifier. When the integrated waveform was pulse‐synchronous and the signal‐to‐noise ratio was high enough (>3 signal‐to‐noise ratio), the waveform was accepted as MSNA burst (Vallbo et al., 1979). These bursts were further evaluated by software that identified bursts based upon fixed criteria, including an appropriate latency following the R‐wave of the electrocardiogram (Vallbo et al., 1979). the MSNA bursts were expressed as the number per minute (burst frequency, BF) (bursts/minute) and the number per 100 heartbeats (burst incidence, BI) (bursts/100 heartbeats).

### 2.3 Statistical analysis

All values are presented as means ± standard deviation. Statistical analyses were performed using SPSS software (version 25, IBM Corp., Armonk, NY, USA). Interclass correlation coefficients (ICC) of two measurements (i.e., ICC (1,1)) for API and AVI were calculated to verify the reliability (Landis & Koch, 1977; Portney & Watkins, 2009). The unpaired *t*‐test was used to compare differences between the healthy and HT groups. Welch's *t*‐test was applied if the variance was heteroscedastic. Propensity score (PS) matching by age and sex was performed to adjust for the differences in the baseline characteristics between the groups. Univariate linear regression analysis was performed to evaluate the relationships between the parameters (age, BMI, SBP, HR, and MSNA) and the AVI or API. Multivariate linear regression analysis was performed to determine the strength and independence of the correlations between the parameters and the AVI or API. The variables of *p* < 0.10 in the univariate analyses were entered into the multivariate analyses. A two‐sided *p* < 0.05 was considered significant.

## 3 RESULTS

### 3.1 Comparison between groups

The baseline characteristics of the participants in each group are presented in Table 1. The participant age (62.0 ± 13.6 vs. 40.1 ± 15.2 years, *p* < 0.001) and proportion of females (*p* = 0.017) were significantly higher in the HT group than in the healthy group.

**TABLE 1:** Age, gender, and hemodynamic characteristics of the groups

	Healthy subjects (*N* = 40)	HT patients (*N* = 40)	*p*
Age (years)	40.1 ± 15.2	62.0 ± 13.6	<0.001
Male/Female	32/8	22/18	0.017
BMI (kg/m^2^)	23.2 ± 3.5	23.9 ± 4.2	0.386
SBP (mmHg)	113.7 ± 9.2	141.6 ± 17.0	<0.001
DBP (mmHg)	67.1 ± 9.1	81.6 ± 14.1	<0.001
HR (beats/min)	67.6 ± 11.5	73.5 ± 11.9	0.026
BF (bursts/min)	23.5 ± 6.9	34.4 ± 11.4	<0.001
BI (bursts/100 heartbeats)	37.1 ± 9.5	49.7 ± 13.2	<0.001
*Medications N (%)*			
CCB	0 (0)	15 (37.5)	
ARB/ACE‐I	0 (0)	15 (37.5)	
Beta‐blocker	0 (0)	7 (17.5)	
Alpha‐blocker	0 (0)	0 (0)	
Diuretics	0 (0)	2 (5)	
MR antagonist	0 (0)	1 (2.5)	

Note

Values are mean ± SD or *N* (%).

Abbreviations: ACE‐I, angiotensin‐converting enzyme inhibitors; API, arterial pressure‐volume index; ARB, angiotensin receptor blocker; AVI, arterial velocity pulse index; BF, burst frequency; BI, burst incidence; BMI, body mass index; CCB, calcium channel blocker; DBP, diastolic blood pressure; HR, heart rate; MR, mineralocorticoid receptor; SBP, systolic blood pressure.

**FIGURE 1:**
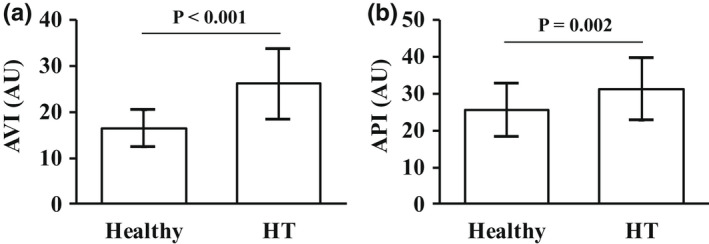
The AVI and API in healthy subjects and HT patients. Comparisons of the AVI (a) and API (b) between the healthy and HT groups. Abbreviations: AVI, arterial velocity pulse index; AU, arbitrary unit; API, arterial pressure‐volume index; HT, hypertension

SBP and DBP were significantly higher in the HT group than in the healthy group (both *p* < 0.001). HR was also significantly higher in the HT group than in the healthy group (73.5 ± 11.9 vs. 67.6 ± 11.5 beats/min, *p* = 0.026). MSNA was significantly higher in the HT group than in healthy group (BF, 34.4 ± 11.4 vs. 23.5 ± 6.9 bursts/min, *p* < 0.001; BI, 49.7 ± 13.2 vs. 37.1 ± 9.5 bursts/100 heartbeats, *p* < 0.001). As shown in Figure 1, the AVI and API were significantly higher in the HT group than in the healthy group (AVI, 26.1 ± 7.6 vs. 16.5 ± 4.0 arbitrary unit, *p* < 0.001; API, 31.2 ± 8.6 vs. 25.5 ± 7.2 arbitrary unit, *p* = 0.002) (Figure 1). The ICC (1.1) of AVI was 0.86 and the ICC of API was 0.72. In the PS matching by age and sex, a caliper size (tolerance) of 0.077, a quarter of a standard deviation of the logit of the PS, was applied to the matching (Cochran & Rubin,; Rosenbaum & Rubin, 1985). As a result, 18 subjects in each group were matched (Table 2). Even after matching by age and sex, SBP and DBP were higher in the HT group than the healthy group (both, *p* < 0.001). The AVI and API were significantly higher in the HT group than in the healthy group (AVI, 23.8 ± 6.1 vs. 18.2 ± 4.9, *p* = 0.004; API, 31.2 ± 8.6 vs. 25.5 ± 7.2, *p* = 0.028).

**TABLE 2:** Age, gender, and hemodynamic characteristics of the groups matched by age and sex

	Healthy subjects (*N* = 18)	HT patients (*N* = 18)	*p*
Age (years)	51.7 ± 14.9	53.2 ± 14.4	0.760
Male/female	13/5	11/7	0.480
BMI (kg/m^2^)	24.4 ± 4.0	24.0 ± 4.3	0.777
SBP (mmHg)	112.2 ± 7.5	141.0 ± 14.0	< 0.001
DBP (mmHg)	66.8 ± 9.1	85.9 ± 14.8	< 0.001
HR (beats/min)	68.1 ± 12.6	74.1 ± 11.4	0.143
AVI (AU)	18.2 ± 4.9	23.8 ± 6.1	0.004
API (AU)	25.5 ± 7.2	31.2 ± 8.6	0.028
BF (bursts/min)	26.3 ± 7.3	32.9 ± 13.3	0.077
BI (bursts/100 heartbeats)	41.1 ± 9.6	45.8 ± 12.1	0.208

Note

Values are mean ± SD. Subjects in the healthy and HT groups were matched according to age and BMI by their propensity score using a tolerance value of 0.077.

Abbreviations: API, arterial pressure‐volume index; AU, arbitrary unit; AVI, arterial velocity pulse index; BF, burst frequency; BI, burst incidence; BMI, body mass index; DBP, diastolic blood pressure; HR, heart rate; SBP, systolic blood pressure.

### 3.2 Correlation between AVI and MSNA

**FIGURE 2:**
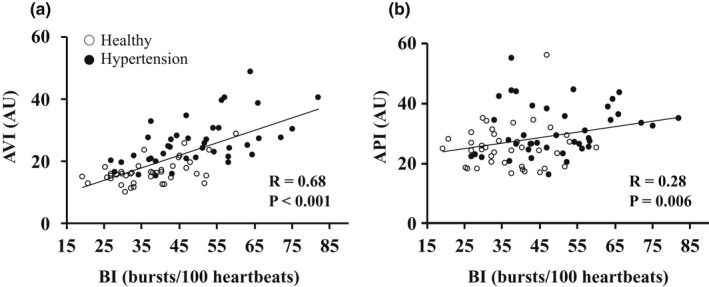
The relationships of MSNA with the AVI or API in all subjects. Relationships between MSNA and the AVI (a) and between MSNA and the API (b) in all subjects. *Note*: White circles indicate healthy subjects, and black circles indicate patients with HT. Abbreviations: AVI, arterial velocity pulse index; AU, arbitrary unit; API, arterial pressure‐volume index; BI, burst incidence: MSNA, muscle sympathetic nerve activity; R, correlation coefficient

**FIGURE 3:**
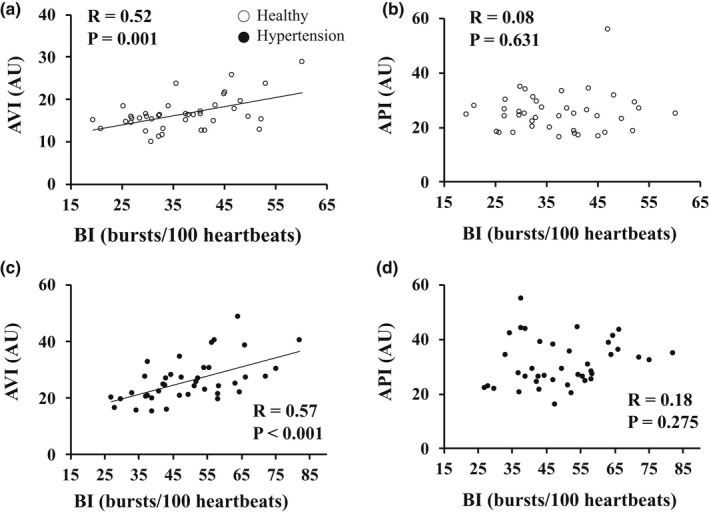
The relationships of MSNA with the AVI or API in each group. Relationships between MSNA and the AVI (a) and between MSNA and the API (b) in healthy subjects. Relationships between MSNA and the AVI (c) and between MSNA and the API (d) in patients with HT. Abbreviations: AVI, arterial velocity pulse index; AU, arbitrary unit; API, arterial pressure‐volume index; BI, burst incidence; MSNA, muscle sympathetic nerve activity; HT, hypertension; R, correlation coefficient

The results of univariate regression analysis between MSNA and the AVI or API in all subjects are shown in Figure 2. MSNA (BI) was significantly correlated with the AVI (*R* = 0.68, *p* < 0.001). MSNA was also correlated with the API, but the correlation coefficient was not high (R = 0.28, *p* = 0.006). The same relationship was observed between BF and AVI (*R* = 0.58, *p* < 0.001). In univariate analysis, age and SBP were also correlated with the AVI, and SBP was correlated with the API (Table 3). In multivariate analysis, age, SBP, and MSNA (BI) were independently correlated with the AVI, and the standardized coefficient in MSNA was the highest among them (*ß *= 0.34, *p* = 0.001). SBP was independently correlated with the API, but MSNA was not correlated with API in multivariate analysis (Table 3). Figure 3 shows the relationship between MSNA (BI) and the AVI or the API in each group. BI was significantly correlated with the AVI (*R* = 0.52, *p* = 0.001) (Figure 3a), whereas BI was not correlated with the API (*R* = 0.08, *p* = 0.631) (Figure 3b) in healthy subjects. The same relationship was observed between BF and the AVI or API (BF vs. AVI, *R* = 0.39, *p* = 0.012; BF vs. API, *R* = 0.05, *p* = 0.779). BI was significantly correlated with the AVI in patients with HT (*R* = 0.57, *p* < 0.001) (Figure 3c) but was not correlated with the API (*R* = 0.18, *p* = 0.275) (Figure3d). The same relationship was observed between BF and the AVI or API in patients with HT (BF vs. AVI, *R* = 0.39, *p* = 0.012; BF vs. API, *R* = 0.05, *p* = 0.779).

**TABLE 3:** Univariate and multivariate regression analyses of the associations of the AVI or API with other parameters in all subjects

	AVI (AU)					API (AU)				
	Univariate		Multivariate			Univariate		Multivariate		
	*R*	*p*	*ß*	*p*	*VIF*	*R*	*p*	*ß*	*p*	*VIF*
Age (years)	0.60	<0.001	0.32	<0.001	1.34	0.12	0.150			
BMI (kg/m^2^)	−0.10	0.397				0.01	0.938			
SBP (mmHg)	0.62	<0.001	0.31	0.001	1.53	0.48	<0.001	0.48	<0.001	1.51
HR (beats/min)	0.03	0.384				−0.05	0.323			
BI (bursts/100 heartbeats)	0.68	<0.001	0.34	0.001	1.76	0.28	0.006	0.002	0.984	1.51

Note

The variables entered into the multivariate regression analysis were those with *p* < 0.10 in the univariate analyses.

Abbreviations: API, arterial pressure‐volume index; AU, arbitrary unit; AVI, arterial velocity pulse index; BI, burst incidence; BMI, body mass index; DBP, diastolic blood pressure; HR, heart rate; R, correlation coefficient; SBP, systolic blood pressure; ß, standardized partial regression coefficient; VIF, variance inflation factor.

## 4 DISCUSSION

The novel findings of the present study are that (1) the AVI and API, which are new arterial stiffness indices, were significantly increased in patients with HT compared with healthy individuals even after adjusting for age and sex, and (2) MSNA was independently correlated with the AVI but was not correlated with the API in healthy subjects and patients with HT.

### 4.1 The relationships between the AVI or API and hemodynamic characteristics

The finding that the average AVI and API were significantly higher in the HT group than in the healthy group is consistent with the evidence of high arterial stiffness in patients with HT (Safar, 2018). Regarding linear relationships, age and SBP were significantly correlated with the AVI, and SBP was significantly correlated with the API, which was consistent with previous reports (Komatsu et al., 2017; Okamoto et al., 2016). Not the arterial stiffness but the effect of age/BP on AVI/API can't completely be excluded since other established indices of arterial stiffness (e.g., PWV, carotid artery ultrasound) were not measured in this study. To clarify this issue, further studies comparing AVI/API to other arterial stiffness indices (e.g., cf‐PWV, carotid artery ultrasound) with age and BP‐matched subjects are expected.

### 4.2 Relationships between MSNA and AVI or API

Arterial stiffness is composed of both structural remodeling (e.g., degradation of elastin, increased collagen deposition) induced by the accumulation of mechanical stress and vascular smooth muscle tone (Nardone et al., 2020). MSNA innervates resistance vessels within skeletal muscle and regulates vascular tone (Shoemaker, 2017; Wallin & Charkoudian, 2007). Therefore, it is understandable that MSNA is correlated with arterial stiffness indices, as shown in previous reports (Boutouyrie et al., 1994; Casey et al., 2011; Dinenno et al., 2000; Grassi et al., 1995; Hart et al., 2013; Holwerda et al., 2019; Kosch et al., 2002; Millar et al., 2019; Swierblewska et al., 2010; Tanaka et al., 2017). In the present study, as we hypothesized, the AVI was independently correlated with MSNA. While, the API was not independently associated with MSNA, which was different from our hypothesis. API reflects the stiffness of only the brachial artery region, where the cuff was attached (Komine et al., 2012). In the previous studies, MSNA was related to the stiffness of peripheral arteries, including the brachial artery (Boutouyrie et al., 1994; Dinenno et al., 2000; Grassi et al., 1995; Kosch et al., 2002). On the other hand, the effect of sympathetic activation on carotid‐brachial PWV was relatively less compared with the effect on cf‐PWV (Holwerda et al., 2019), and less *α*‐adrenergic receptor density/sensitivity in the brachial region compared with the femoral region was suggested (Fairfax et al., 2013; Holwerda et al., 2019; Pawelczyk & Levine, 1985). Therefore, the API, evaluating only the brachial region of the cuff attached, might not be sensitive enough to evaluate the effect of efferent SNA on arterial stiffness at rest. Future studies evaluating MSNA and API with sympathoexcitation interventions are expected to investigate the effect of MSNA on API.

In contrast to the API, AVI does not focus on the peripheral arterial stiffness itself but on the reflected waves, too (Komatsu et al., 2017; Sueta et al., 2015). High arterial stiffness can cause the high velocity of both forward flow and reflected waves. In this instance, reflected waves are considered an arterial stiffness index (Laurent et al., 2006; O'Rourke & Mancia, 1999). Moreover, wave reflection can also be affected by the vascular tone of small muscular arteries at the reflected site. Thus, reflected waves are considered more strongly affected by peripheral vascular resistance than arterial stiffness itself (Kelly et al., 2001). In fact, the vascular smooth muscle tone was associated with AI, a conventional index of reflected waves, independent from arterial stiffness (i.e., cf‐PWV) (Kelly et al., 2001). As described above, MSNA innervates resistant vessels in muscles (Barretto et al., 2009; Hamaoka, Blaha, et al., 2021), so it is plausible that MSNA was more closely correlated with the index of reflected waves (i.e., AVI) than the index of the brachial artery stiffness (i.e., API).

### 4.3 Advantages of measuring AVI

A clear advantage of measuring AVI is the simplicity of the measurement. Commonly, arterial inflow wave separation analyses are needed to evaluate reflected waves (Mynard et al., 2020). However, the AVI needs only the time differentiation of the waveform and does not need any adding/subtraction processes for the waveform (Mynard et al., 2020), and most calculations undergo automatically high reproducibility (Okamoto et al., 2016). In this study, the reproducibility was high (ICC =0.86) (Landis & Koch, 1977; Portney & Watkins, 2009), consistent with the previous report (Okamoto et al., 2016). Reflected waves would affect central hemodynamics (Komatsu et al., 2017; Sueta et al., 2015), and central hemodynamics has shown independent predictive value for CV events (Weber et al., 2005; Williams et al., 2006). Therefore, the ability to assess the parameter that affects central hemodynamics (i.e., reflected waves) via the simple method is an obvious advantage of AVI.

Although AI had been considered as an established index of reflected waves, the reported relationship between MSNA and AI has been inconsistent (Casey et al., 2011; Hart et al., 2013; Millar et al., 2019; Wakeham et al., 2022). One possible reason is the difference in the characteristics of the participants (e.g., young female (Casey et al., 2011), healthy or heart failure (Millar et al., 2019), athletes, (Wakeham et al., 2022)). Still, even when considering only the healthy subjects, the relationships were not entirely consistent (Casey et al., 2011; Hart et al., 2013; Millar et al., 2019; Wakeham et al., 2022). In a recent study, AI was affected by both cardiac and vascular properties (Heusinkveld et al., 1985). Thus, the understanding that AI is a proxy for the wave reflection magnitude has been questioned (Heusinkveld et al., 1985). Initial forward flow velocity/slope (i.e., dp/dt) calculated from the peripheral artery waveform was suggested to be related to cardiac output (Jansen et al., 2001; Romano & Pistolesi, 2002; Vaal et al., 2005). Although the method measuring the waveform is different, Vf also reflects dp/dt of the initial forward flow (Sueta et al., 2015). As described above method section, the AVI is calculated by dividing the Vr by Vf; hence, theoretically, AVI is adjusted for Vf, which would reflect cardiac output. This adjustment in the AVI calculation might help diminish the effect of cardiac output on AVI values and contributed to the significant relationship between MSNA and AVI in this study. Furthermore studies evaluating AVI and AI in subjects with impaired cardiac function are warranted.

Reflected waves are suggested as an indirect, surrogate measure of arterial stiffness, then in the expert consensus, the measurement of the reflected waves is recommended to be optimally coupled with the measurement of aortic PWV to determine the contribution of arterial stiffness to wave reflections (Laurent et al., 2006). If API could be used, in part, alternatively to PWV as the direct arterial stiffness index, it would support the usefulness of the method that can measure both AVI and API simultaneously, however, further studies are needed for this issue.

### 4.4 Limitations

This study contains several limitations. First, subjects were consecutively included, and the baseline characteristics differed between the groups. The difference in baseline characteristics (e.g., age) in the results cannot be entirely dismissed. To minimize this difference, we performed PS score matching; the results regarding AVI and API remained unchanged after PS matching (the AVI and API were higher in the HT group than the healthy group). In comparison, the group difference in MSNA was weakened by PS matching, which was inconsistent with the previous meta‐analysis result (Grassi et al., 2018). Aging is also a well‐known contributor to increases in MSNA (Keir et al., 2020; Matsukawa et al., 1998; Narkiewicz et al., 2005), and the PS matching decreased the number of subjects. The statistical power was possibly not enough to detect the group difference in MSNA after the matching; however, the tendency was still observed (*p* = 0.08). In multivariate analysis, MSNA was significantly correlated with AVI independently of age. Thus, we believe that our main outcome (significant correlation between MSNA and AVI) was not compromised by age difference. Second, some patients with HT had received medications, which could have affected the results. The number of subjects who received medications was not large (18.8% of all subjects), and despite the difference in medication history, a significant linear relationship between the AVI and MSNA was consistent in both the healthy and HT groups. Third, the number of females was small (20% in the healthy group), and the day in the menstrual cycle or menopause were not investigated in females. Thus, we could not verify the sex differences in this study. Finally, we only evaluated the AVI and API at rest. Examining the effect of sympathoexcitation on these indices is essential to better understand the relationships between these indices and SNA. Further studies on this issue are expected.

### 4.5 Perspective

A feasible method with high reproducibility is needed to detect increased arterial stiffness earlier and evaluate the response to treatments. Our results would support the reliability of AVI by showing the significant association with MSNA. Recently, neuromodulation therapies targeting elevated SNA for drug‐resistant HT (Kiuchi et al., 2019) and heart failure (Zile et al., 2020) have been developed (i.e., renal denervation and baroreflex activation therapy). However, their efficacy is still under discussion (Kiuchi et al., 2019). When applying these treatments to daily clinical practice, a method that can evaluate the effect of the treatment simply and accurately is required. Significant reductions in MSNA, cf‐PWV, and AI by neuromodulation therapies have been reported (Brandt et al., 2012; Hering et al., 2013; Wallbach et al., 2015). However, applying these methods widely in daily clinical practice is still challenging because of their methodological limitations, as described in the above introduction and discussion section. AVI and API may be valuable methods to evaluate the outcome of these treatments because of their simplicity and high reproducibility (Okamoto et al., 2016). Especially, AVI might be a feasible index to assess the effect of neuromodulation therapy on hemodynamics, including SNA (via evaluating the change in arterial reflected waves). Future studies investigating the AVI and API in patients with elevated SNA (e.g., drug‐resistant HT and heart failure) are needed for this issue.

### 4.6 Conclusion

Both AVI and API were increased in patients with HT compared to healthy participants. There is a significant relationship between MSNA and AVI, but not API, which is likely related to the influence of MSNA on arterial wave reflection. Our results indicate that AVI would be a useful index to simply assess vascular smooth muscle tone alternation in daily clinical practice.

## CONFLICT OF INTEREST

The authors declare no competing financial interests.

## AUTHOR CONTRIBUTIONS

Hamaoka T, Murai H, and Takamura M, conceived and designed research; Sugimoto H, Hamaoka T, Murai H, Hirai T, and Mukai Y performed experiments; Sugimoto H, Hamaoka T, and Mukai Y analyzed data; Sugimoto H, Murai H, Kusayama T, Kato T, Takashima S, Usui S, and Takata S recruited subjects; Sugimoto H, Hamaoka T, Murai H, Sakata K, and Kawashiri M interpreted results of experiments; Sugimoto H and Hamaoka T prepared figures; Sugimoto H, Hamaoka T, Murai H, Usui S, and Takamura M drafted the manuscript; All co‐authors approved the final version of the manuscript.
